# Surgical Fixation and Inter-phalangeal Arthrodesis of Symptomatic Non-union of Fracture of a Lesser Toe Distal Phalanx: A Case Report

**DOI:** 10.5704/MOJ.1911.012

**Published:** 2019-11

**Authors:** GL Foo, LHJ Wee

**Affiliations:** Department of Orthopaedic Surgery, Tan Tock Seng Hospital, Singapore

**Keywords:** fixation, trauma, toe, phalanx, non-union

## Abstract

Distal phalanx fractures of the toes are common injuries. The majority of them are treated conservatively with good outcome. We present the case of a painful non-union fracture of the distal phalanx of the 4th toe in a 60-year-old female patient with symphalangism of the 4th and 5th toes. She underwent surgical fixation of the fracture with concomitant inter-phalangeal joint (IPJ) arthrodesis for better stability. A transverse dorsal incision was made just distal to the IPJ to allow preparation of both the fracture site and IPJ. Fibrous tissue at the fracture non-union site was removed and the opposing surfaces drilled with a 0.88mm K-wire. Cartilaginous tissue at the IPJ was removed and similarly drilled with the 0.88mm K-wire. Stabilisation was achieved with a percutaneous headless compression screw. Radiographic union was achieved and the patient had resolution of symptoms 16 weeks after the surgery. The patient continued to be symptom-free at one year follow-up. This is the first case report of a surgically treated symptomatic non-union of distal phalanx fracture of a lesser toe in the literature.

## Introduction

Distal phalanx fractures of the toes are common injuries, forming about 9% of fractures treated in the primary care setting^1^. The vast majority of these fractures heal with non-operative management, typically with good outcomes. We were unable to find any previous report on the surgical management of such fractures that had gone into symptomatic non-union. We present a case of a painful non-union fracture of a 4th toe distal phalanx in a patient with symphalangism of the 4th and 5th toes which was stabilised with a headless compression screw.

## Case Report

**Fig. 1:**
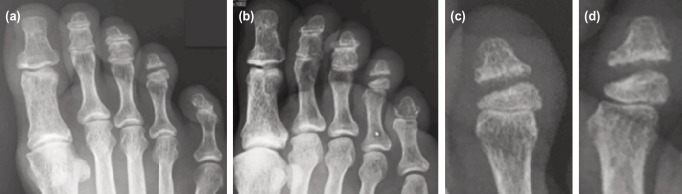
Pre-operative radiographs of fracture non-union of right fourth toe distal phalanx. (a) Dorso-plantar foot view, (b) Oblique foot view, (c) Magnified dorso-plantar fourth toe view, (d) Magnified oblique fourth toe view.

Our patient was a 60-year-old female who sustained the injury when a trolley ran over her right 4th toe. It was a closed injury with no significant deformity. Radiographs showed a distal phalanx oblique fracture (Fig. 1). Incidentally, this patient also had symphalangism involving the middle and distal phalanges of her 4th and 5th toes.

Her past medical history included hyperlipidaemia and lumpectomy for breast cancer. She was also a hepatitis C carrier. She was on regular medication with Rosuvastatin 5mg nightly for the management of hyperlipidaemia. She was a non-smoker.

As the toe alignment and soft tissue condition were satisfactory, the patient was initially treated conservatively with buddy splinting and advised to mainly weight-bear on the heel to avoid loading the forefoot. Serial radiographs at subsequent reviews did not show any signs of union, even after conversion to a short walker boot after three months. At five months after the initial injury, the patient was still symptomatic, especially with prolonged ambulation. The patient worked as a nurse and spent a significant amount of time on her feet at work. Outside of work, the patient was an avid trekker. Having failed conservative management and given her high functional demands, she was counselled regarding open reduction and internal fixation of the fracture. In view of the short proximal fragment, stable fixation of the distal phalanx alone was anticipated to be technically difficult, and she was advised on the option of fusion across the inter-phalangeal joint (IPJ) to improve stability, to which she consented.

**Fig. 2:**
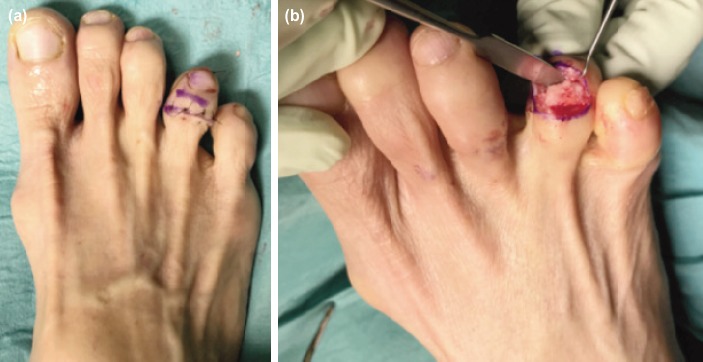
(a) Surgical approach with dorsal transverse incision over right fourth toe distal inter-phalangeal joint. (b) Fibrous tissue at fracture site with sclerotic bone edges.

Subsequently, the patient underwent surgical fixation of the 4th toe distal phalanx fracture with concomitant arthrodesis of the IPJ. A transverse dorsal incision was made just distal to the IPJ to allow preparation of both the fracture site and IPJ surfaces (Fig. 2). The fracture site was mobile with the presence of fibrous tissue and sclerotic bone surfaces, consistent with fibrous non-union (Fig. 2). This was removed and the opposing sclerotic surfaces drilled with a 0.88mm Kirschner wire (K-wire) to optimise bone healing. Cartilaginous tissue at the IPJ was removed in preparation for arthrodesis, and similarly drilled with the 0.88mm K-wire.

The toe was reduced with manual axial compression and stabilised with 2 x 0.88mm K-wires. A stab incision was made at the tip of the toe to allow passage of the K-wires and screw. The K-wires were first inserted in a retrograde fashion via the fracture site out through the tip of the toe, and then passed antegrade through the IPJ. Compression was achieved with a cannulated 20mm percutaneous headless compression screw [Acutrak 2 Micro ®: Acumed, Portland, OR]. The screw had a diameter of 2.5mm at the tip and 2.8mm at the tail. There was already partial resorption of the proximal fragment of the distal phalanx, resulting in a triangular configuration in the axial plane. Due to this configuration, the overall toe alignment was in slight valgus but there was no impingement against the adjacent toes in both flexion and extension.

**Fig. 3:**
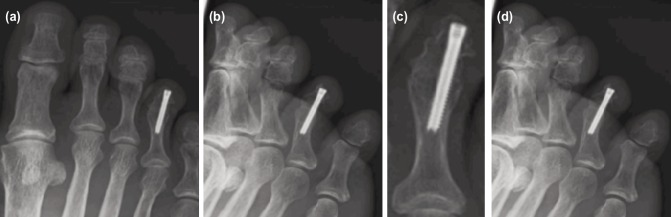
Post-operative radiographs at six months, showing union of right fourth toe distal phalanx fracture and inter-phalangeal fusion. (a) Dorso-plantar foot view, (b) Oblique foot view, (c) Magnified dorso-plantar fourth toe view, (d) Magnified oblique fourth toe view.

Skin closure was with synthetic, non-absorbable monofilament 4-0 suture. The patient was kept on heel weight bearing with a forefoot-offloading shoe. Her surgical site sutures were removed at two weeks and the wound healed without any complication. She was taken off the forefoot off-loading shoe at ten weeks and allowed progressive weight-bearing as tolerated on her forefoot. At review six months after surgery, she reported that there was no pain in her toe. She was coping well at work, and had returned to her trekking activities without any problems. The patient also reported no limitations in her choice of footwear. Radiographs of her toe showed union of the fracture (Fig. 3). At final review one year after surgery, she continued to be symptom-free.

## Discussion

The majority of distal phalanx fractures of the toes are treated conservatively. The threshold for satisfactory alignment and conservative management includes: angulation of less than 20 degrees in the sagittal plane, less than 10 degrees in the axial plane and less than 20 degrees in the coronal plane (rotational).

Fractures with satisfactory alignment can be stabilised with buddy splints and immobilised with rigid-sole shoes^2^. These fractures usually heal with good outcomes. In rare cases where healing does not occur and the fracture goes into non-union, surgery can be offered to patients who are symptomatic.

There is a paucity of literature on the surgical management of this condition in the toes. We tapped on the literature involving distal phalangeal fractures of the fingers to guide our surgical planning^3^. Due to the small size of the distal phalanx fragments and narrow soft tissue envelope, fixation options were limited to K-wires (0.8 to 1.2mm), cortical screws (1.3 to 1.5mm) and headless compression screws (2.5mm). Cortical screw fixation was associated with greater IPJ range of motion compared with K-wire fixation, but required implant removal in half of the cases due to the prominence of the screw head^3^. There are also newer expandable implants such as the X-Fuse® and Smart Toe® [Stryker, Kalamazoo, MI] which have shown promising results in recent reports^4, 5^.

We decided on the headless compression screw as we could obtain adequate bone stock by performing a concomitant IPJ fusion which would provide the most stable fixation in our high-activity patient, compared to K-wires and cortical screws. The headless compression screw provided double compression across both the fracture and fusion sites. The cannulated system also allowed easy instrumentation and passage of the screw. This surgical approach enabled us to achieve successful fracture union and IPJ arthrodesis, with satisfactory functional outcome for our patient.

This case report illustrates the successful treatment of a non-united and painful fracture of the distal phalanx of the toe, using a headless compression screw to achieve stable fracture fixation with fusion across the IPJ.
